# Improving Deep Brain Stimulation Electrode Performance *in vivo* Through Use of Conductive Hydrogel Coatings

**DOI:** 10.3389/fnins.2021.761525

**Published:** 2021-11-05

**Authors:** Tomoko Hyakumura, Ulises Aregueta-Robles, Wenlu Duan, Joel Villalobos, Wendy K. Adams, Laura Poole-Warren, James B. Fallon

**Affiliations:** ^1^The Bionics Institute of Australia, East Melbourne, VIC, Australia; ^2^Department of Medical Bionics, The University of Melbourne, Parkville, VIC, Australia; ^3^Graduate School of Biomedical Engineering, The University of New South Wales, Sydney, NSW, Australia; ^4^Tyree Foundation Institute of Health Engineering, The University of New South Wales, Sydney, NSW, Australia

**Keywords:** conductive hydrogel, deep brain stimulation, neural stimulation, electrode coating, tissue response, electrical properties

## Abstract

Active implantable neurological devices like deep brain stimulators have been used over the past few decades to treat movement disorders such as those in people with Parkinson’s disease and more recently, in psychiatric conditions like obsessive compulsive disorder. Electrode-tissue interfaces that support safe and effective targeting of specific brain regions are critical to success of these devices. Development of directional electrodes that activate smaller volumes of brain tissue requires electrodes to operate safely with higher charge densities. Coatings such as conductive hydrogels (CHs) provide lower impedances and higher charge injection limits (CILs) than standard platinum electrodes and support safer application of smaller electrode sizes. The aim of this study was to examine the chronic *in vivo* performance of a new low swelling CH coating that supports higher safe charge densities than traditional platinum electrodes. A range of hydrogel blends were engineered and their swelling and electrical performance compared. Electrochemical performance and stability of high and low swelling formulations were compared during insertion into a model brain *in vitro* and the formulation with lower swelling characteristics was chosen for the *in vivo* study. CH-coated or uncoated Pt electrode arrays were implanted into the brains of 14 rats, and their electrochemical performance was tested weekly for 8 weeks. Tissue response and neural survival was assessed histologically following electrode array removal. CH coating resulted in significantly lower voltage transient impedance, higher CIL, lower electrochemical impedance spectroscopy, and higher charge storage capacity compared to uncoated Pt electrodes *in vivo*, and this advantage was maintained over the 8-week implantation. There was no significant difference in evoked potential thresholds, signal-to-noise ratio, tissue response or neural survival between CH-coated and uncoated Pt groups. The significant electrochemical advantage and stability of CH coating in the brain supports the suitability of this coating technology for future development of smaller, higher fidelity electrode arrays with higher charge density requirement.

## Introduction

Deep brain stimulation (DBS) uses electrodes inserted into specific regions of the brain to deliver therapeutic electrical stimulation, such as those targeting the subthalamic nucleus (STN) in treating Parkinson’s disease. Indeed, DBS has been used to treat an expanding number of indications over the past decade, including both neurological and psychiatric disorders (McDermott, 2016; Youngerman et al., 2016). Contemporary DBS devices utilise large ring electrodes of approximately 1.3 mm diameter (6 mm^2^ surface area) that can result in a significant risk of damage to brain tissue during insertion (Ory-Magne et al., 2007; Boviatsis et al., 2010; Morishita et al., 2013). In addition, undesirable off-target activation due to spread of stimulation from large electrodes can be an issue (Anderson et al., 2019; Nguyen et al., 2019). Smaller electrodes have been developed to reduce insertion trauma and improve targeting (Villalobos et al., 2020), however, electrode size is limited by the electrochemical properties of the electrode material (Merrill et al., 2005; Cogan, 2008; Wellman et al., 2018). Advancement of electrode design therefore requires advancement in electrode materials. For example, decreasing electrode size for higher fidelity recording and stimulation results in higher charge densities which can impact on device performance.

There are many new electrode and coating materials currently being investigated to safely increase charge injection capacity, however, performance of many coatings can degrade *in vivo* (Patel et al., 2016; Ji et al., 2019; Dalrymple et al., 2020; Welle et al., 2020). The aim of this study was to examine the stability, electrochemical performance and tissue response of a new, lower swelling conductive hydrogel (CH) coating over 8 weeks implantation in the brain. CHs as coatings for traditional metal electrode materials such as platinum (Pt) have been shown to improve the mechanical stability of highly conductive polymers (Green et al., 2012b) and to improve the electrode performance by increasing the effective surface area available for charge transfer and reducing the mechanical mismatch at the electrode-tissue interface (Aregueta-Robles et al., 2014).

Previous *in vitro* studies demonstrated that a CH coating consisting of poly(ethylene dioxythiophene) (PEDOT) deposited in a poly(vinyl alcohol) (PVA) hydrogel modified with taurine and methacrylate functional groups (PVA-Taurine), lowers electrode impedance and increases charge storage capacity (CSC) and CIL compared to smooth Pt electrodes (Dalrymple et al., 2019). The improvement was maintained during accelerated aging equivalent to 1.6 years of use. A 5 weeks *in vivo* study of PVA-Taurine CH-coated electrodes implanted into rat cochlea demonstrated similar improvements in electrode performance compared with standard smooth Pt (Dalrymple et al., 2020). There was no observed impact on evoked response or neural survival. However, tissue response tended to be greater in the CH group compared with the Pt group, which may have been associated with loss of material from the CH coating (Dalrymple et al., 2020). In other studies, both Pt and silicone particles have been observed in tissue surrounding cochlear implants and increased tissue responses are associated with the presence of such particles (O’Malley et al., 2017; Simoni et al., 2020; Shepherd et al., 2021).

Loss of coating materials can be a result of poor adhesion to the substrate, poor cohesion or high friction causing fracture of the material during handling and insertion. CH adhesion to Pt relies on conductive polymer pre-layer coating of Pt substrates prior to CH fabrication (Goding et al., 2017) and the results from *in vivo* implantation suggested that the coatings remained largely intact (Dalrymple et al., 2020). However, the latter studies were conducted in a cochlear implant model in which arrays were inserted into the fluid-filled cavity of the cochlea with minimal direct contact with target neurons. In contrast DBS electrodes are inserted into brain tissue and directly contact the tissue during insertion resulting in very different mechanical stresses. Moreover, the hydrogel component of the CH system used in previous studies inherently swells, which increases the size of the electrode, thus compressing and distancing from surrounding tissue. Any swelling of the coating may also compromise its mechanical stability upon implantation. In this study, we engineered a new CH formulation tailored with reduced swelling and evaluated the effect of insertion into brain tissue on both electrochemical performance and on coating cohesion and stability during insertion, implantation, and removal.

The new CH coating designed had lower swelling properties than the previously studied variants. The stimulating and recording performance of the CH-coated electrodes was examined in model brain tissue *in vitro* as well as *in vivo* over 8 weeks in a rat DBS model, which places the CH coating directly adjacent to target neurons, different to the fluid-filled cochlea studied previously (Dalrymple et al., 2020). As previous studies with higher swelling CH demonstrated an increased tissue response compared with Pt in the rat cochlea, the tissue response at the electrode-tissue interface in the brain was also systematically evaluated. The study hypothesised that a higher stability CH coating will improve electrochemical performance and not adversely affect brain tissue response to implanted electrodes compared with uncoated control Pt electrodes.

## Materials and Methods

### Materials

All reagents and materials were purchased from Sigma unless stated otherwise. PVA (∼16 kDa, 98% hydroxylation) was used for synthesis and fabrication of methacrylated PVA (PVA-MA) macromers and methacrylated/taurine-conjugated (PVA-Tau) macromers as previously described by Goding et al. (2017). Hydrogel polymerisation was performed with the photo-initiator 2-Hydroxy-4′-(2-hydroxyethoxy)-2-methylpropiophenone (Irgacure 2959). The 3,4-ethylenedioxythiophene (EDOT) monomer was used for electrodeposition of the conductive polymer (PEDOT) doped with either sodium p-toluenesulfonate (pTS) or taurine. Model electrodes (MEs) were fabricated with 99.95% Pt wire (Ø = 0.76 mm ± 0.01 mm, 99.95% purity, Surepure Chemetals) and with medical grade poly (dimethyl siloxane) (PDMS) tubing (Point Medical, 0.94 mm in outer diameter, 0.51 mm in inner diameter). The vapour degassing solvent (Lenium^TM^, Novaline) was used for expanding the PDMS tubing. Pt DBS arrays modified for rat animal models were manufactured at the Bionics Institute, Australia. Agarose was used for fabrication of brain tissue *in vitro* models.

### Tailoring Swelling Behaviour of Conductive Hydrogel Electrode Coatings

To control the swelling behaviour of hydrogels used for the fabrication of CH coatings, PVA was chemically modified with 5, 10, and 15 MA groups per chain (theoretical), labelled LOW-MA, MED-MA, and HIGH-MA, respectively. As detailed in Table 1, to vary the taurine content, the PVA-MA macromer formulations were co-polymerised with the previously reported PVA-Tau by Goding et al. (2017). PVA-Tau was the positive, higher swelling control, and comprised PVA modified with ∼6-MA groups/PVA chain for network crosslinking and ∼20-taurine groups/PVA chain required for PEDOT electrodeposition (Dalrymple et al., 2019). All hydrogel test solutions were prepared by dissolving the PVA macromers at a final concentration of 20 wt% in phosphate buffer solution (PBS, pH = 7.4), including the photo-initiator Irgacure 2959 (0.1 wt%). The solution was heated at 80°C, homogenised via vortex agitation and cooled to room temperature prior to polymerisation and testing. Dimensional changes in hydrogel formulations were studied through swelling studies following previously reported methods (Aregueta-Robles et al., 2018) as well as *via* measuring changes in diameter (Ø) with digital callipers. See Supplementary Material for detailed methodology.

**TABLE 1:** PVA hydrogel macromer test formulations used for assessing the effect of MA functionalisation and taurine content on hydrogel swelling behaviour.

**MA groups per PVA chain**	**Hydrogel composition (wt%)**		**PVA-Tau content (%) in co-polymer**	**ID Label**	**CH Formulation**
	**PVA-MA**	**PVA-Tau**			
6	–	20	100	PVA-Tau (positive control)	PEDOT/PVA-Tau (positive control)
6 (low)	20	–	0	LOW-MA 0%	PEDOT/LOW-MA 0%
	15	5	25	LOW-MA 25%	PEDOT/LOW-MA 25%
	10	10	50	LOW-MA 50%	PEDOT/LOW-MA 50%
11 (medium)	20	–	0	MED-MA 0%	Not applicable (NA)
	15	5	25	MED-MA 25%	PEDOT/MED-MA 25%
	10	10	50	MED-MA 50%	PEDOT/MED-MA 50%
16 (high)	20	–	0	HIGH-MA 0%	NA
	15	5	25	HIGH-MA 25%	PEDOT/HIGH-MA 25%
	10	10	50	HIGH-MA 50%	PEDOT/HIGH-MA 50%

*MA groups as determined by NMR.*

### Electrochemical Performance of Conductive Hydrogel Formulations as Coatings on Model Electrodes

The electrochemical performance of CH formulations shown in Table 1 were evaluated using MEs fabricated in house (see Supplementary Material for fabrication of CH coated MEs). A single unblended PVA-MA formulation without taurine (0% PVA-Tau) with low methacrylate was included as a negative control (LOW-MA 0%). Swelling of different CH formulations was compared and the most dimensionally stable variants identified. To assess the electrical performance of coated MEs, the polarisation impedance (Zp) and CSC were determined *via* electrochemical impedance spectroscopy and cyclic voltammetry, respectively, as detailed in the *In vitro* electrochemical testing regime.

### Conductive Hydrogel Coating

MEs and DBS electrode arrays were coated following previously reported procedures (Goding et al., 2017). First, a pre-layer coating of PEDOT doped with pTS was electrically deposited on the electrodes using a potentiostat (eDAQ Pty. Ltd., NSW, Australia) *via* a two-electrode cell setup. The PEDOT/pTS monomer solution consisted of 0.1 M EDOT and 0.05 M pTS dissolved in 50% (v/v) acetonitrile solution, which was deposited at 1 mA/cm^2^. Following deposition, electrodes were washed with deionised water to remove excess monomer solution and dried in a laminar flow cabinet overnight. MEs were coated with CH formulations with varied degrees of swelling characteristics (Table 1). DBS arrays were coated with the CH formulation identified with the lowest degree of swelling and maintained enhanced electrical properties as well as with the previously reported high-swelling variant (PVA-Tau). For simplicity, PEDOT deposited DBS arrays with the low swelling formulation and high swelling formulation were referred to as “LS” and “HS,” respectively. Coated MEs and DBS arrays were soaked in PBS for 24 h to remove unreacted products and left to dry at least for 24 h in a laminar flow cabinet before sterilisation. CH-coated DBS arrays were ethylene oxide (ETO)-sterilised at the Prince of Wales Hospital (Sydney, NSW, Australia).

### Fabrication of Rat Deep Brain Stimulation Electrodes

**FIGURE 1:**
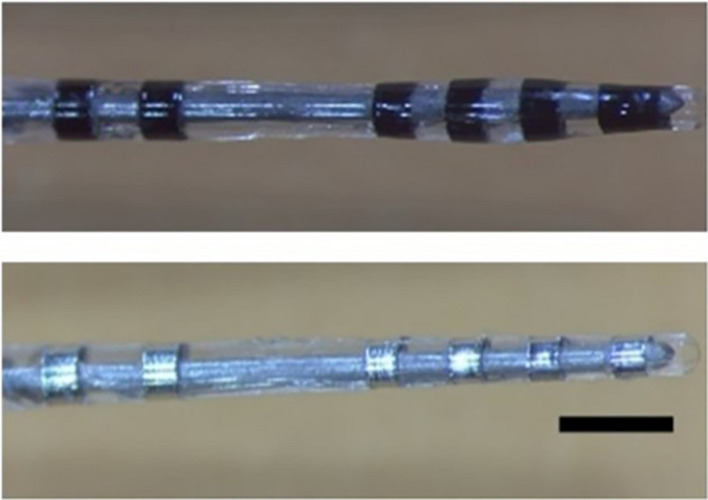
Rat DBS electrode arrays, uncoated (top) and CH-coated (bottom) (scale bar, 1 mm).

Electrode arrays with six electrodes designed to be used for DBS targeting the STN of the rat were made in the electrode fabrication facility at the Bionics Institute, Melbourne, VIC, Australia. They were made up of Pt (99.95%) ring electrodes and wires and medical-grade silicone. The diameter of the ring electrodes were 0.39, 0.41, 0.43, 0.45, 0.55, and 0.57 mm from E1 (most distal) to E6 (most proximal), and all of them were approximately 0.3 mm long. Electrodes 1–4 were spaced roughly 0.77 mm apart from each other (centre to centre). Electrodes 5 and 6 were used as reference electrodes and placed approximately 3.5 mm above E4 (Figure 1).

### Effect of Electrode Insertion on the Coating Stability of Conductive Hydrogel Formulations

**FIGURE 2:**
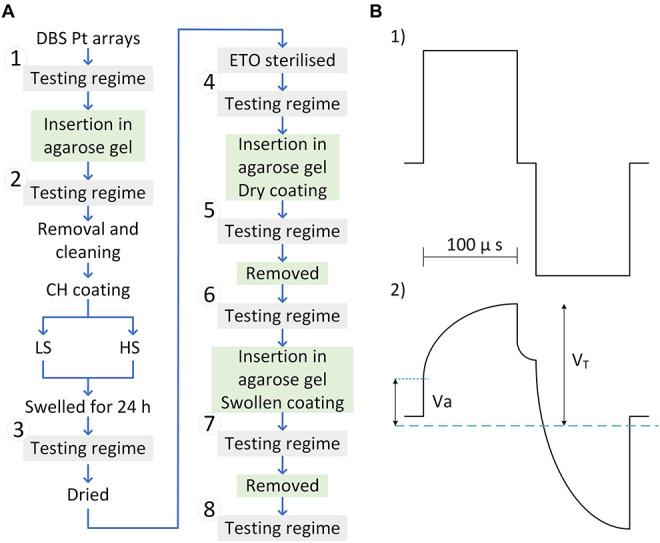
**(A)** Flow chart of tests and treatment performed on DBS arrays. Four arrays were tested in total, two coated with the low swelling formulation (LS), and two coated with the high swelling formulation (HS). The electrochemical performance of uncoated DBS arrays was assessed before and after insertion into the agarose gel to generate baseline Pt Data (1–2). Arrays were then CH-coated and swollen to provide baseline CH properties (3). Following ethylene oxide sterilisation (4) the in-agarose gel testing consisted of assessing changes in electrochemical properties following insertion of dry and swollen CH-coated electrodes in agarose gels (5 and 7) compared to baseline (3). Arrays were removed from the agarose gel and assessed in PBS (6 and 8). **(B)** Biphasic electrode pulse (top) and voltage transient of Pt in reference to Ag/AgCl electrode (bottom).

Deep brain stimulation arrays were cleaned chemically, mechanically, and electrically before electrochemical testing and hydrogel coating as previously outlined (Aregueta-Robles et al., 2020). Electrode coating stability was assessed *via* insertion in an agarose gel model with equivalent mechanical properties to brain tissue (Chen et al., 2004). Agarose was dissolved in MilliQ water (0.6 w/v%) at 100°C for 5 min and allowed to cool to 30–35°C. Once cooled, the solution was poured into cylinder-shaped (h = 10 mm, Ø = 10 mm), 3D printed poly (lactic acid) moulds. Gels were cured at 4°C overnight and allowed to swell in PBS for 24 h prior to experimentation with DBS arrays. Electrode arrays were fixed onto an adapted micromanipulator to control electrode insertion in 0.6% agarose gels at 2–6 mm/s. The coating stability was evaluated *via* assessing electrochemical properties as described in the *in vitro* electrochemical testing regime stage and illustrated in the flow chart in Figure 2A. To assess changes in electrical properties due to the agarose interface, uncoated electrodes were also assessed before and after insertion in the agarose gel. Following a second round of cleaning, arrays were CH-coated with either the HS (*n* = 2) or the LS (*n* = 2). Coated arrays were placed in PBS for removal of sol fraction (24 h), rinsed with MilliQ water, and allowed to dry for 2 h in a laminar flow cabinet. The testing regime was performed following CH fabrication in PBS, which was used as a baseline to assess changes following ETO sterilisation and upon insertion in agarose gels. To study the potential impact of the CH-coating swelling condition on its stability during insertion, the arrays were inserted with the coating dry (named “Inserted Dry”), removed, then re-swollen for 24 h in PBS, and inserted in a freshly made agarose gel (named “Inserted Swollen”) and removed again. The testing regime was performed following each step. The insertion and removal of DBS arrays in agarose gels was recorded *via* a commercial camera mounted to the micromanipulator for visual assessment of the coatings during insertion and upon removal (videos included in Supplementary Material). For consistency, CH-coated arrays were inserted with dry coatings in animal implantation studies.

### *In vitro* Electrochemical Testing Regime

Electrochemical impedance spectroscopy (EIS) and CV were performed using a potentiostat (CH Instruments, Inc.). EIS was performed within 1 Hz–100 kHz frequency range (AC peak to peak = 30 mV). To provide a resolved parameter that accounts for the cumulative effect of the frequency on the impedance, the polarisation impedance “*Z*_*P*_” was calculated *via* the integral of the impedance spectra as described in Eq. 1. As described by the Kramers–Kroning (K-K) relations, fmax is the frequency when the complex impedance reaches a maximum. For simplicity, *f*_*max*_ and *f*→ were taken at 1 Hz and at 100 kHz, respectively.


(1) $$Z_{P}=\frac{9.2}{\pi }\,\int_{f_{m\,a\,x}}^{f\to \infty }Z^{\prime \prime }\,dl\,o\,g\,f$$

Charge storage capacity was determined by cycling a potential between −0.6 and 0.8 V applied to the working electrode at a scan rate of 150 mV/s for five cycles. The cathodic area under the CV curve was calculated. The presented CSC values represent the mean area from cycles 3–5. The CIL and end-of-phase [voltage transient (VT)] impedance were assessed *via* the VT response that was obtained from delivering biphasic charge-balanced pulses using a current-controlled source (Figure 2B-1). VTs were measured *via* an isolated battery-operated oscilloscope. The characteristic voltage response of Pt electrodes to biphasic current pulses is represented in Figure 2B-2 consisting of an immediate rise in voltage (*Va*), followed by a slow transient associated with the polarisation resistance of the electrode. *V*_*T*_ represents the peak voltage measured at the end of the cathodic phase. The VT impedance was calculated following Ohm’s law with a pulse amplitude of 100 mA and phase width of 100 ms.

The polarisation voltage (*Vp = V_*T*_* − *Va*) was used for calculation of the CIL, defined as the threshold charge (*Q = I* × *t*) before water electrolysis. The CIL-current “*I*” was obtained by increasing the current amplitude of a 100 μs pulse from 100 μA until recording a Vp = 0.6 V or up to the maximum current output of the stimulator (16.5 mA).

Electrochemical measurements were performed with a 3-cell set up consisting of a Pt counter electrode (E5 and E6 shorted together), a Ag| AgCl reference electrode and the working electrode of interest from the arrays.

### Animals

Sixteen male Sprague-Dawley rats from Animal Resource Centre, Western Australia were used for this study. They were 10–11 weeks of age at the time of surgery. Procedures were approved by St Vincent’s Hospital Animal Research Committee (project ethics #010/19 and #009/20) and complied with the Australian Code for the Care and Use of Animals for Scientific Purposes (National Health and Medical Research Council of Australia) and the Prevention of Cruelty to Animals (1986) Act. Rats were housed individually after DBS electrode array implantation to avoid damage to the percutaneous connector. Housing conditions were otherwise standard, with *ad libitum* access to standard chow and water, and 12 h light and dark cycle.

### Implantation Surgery

Surgery was performed under aseptic conditions. Rats were anaesthetised with isoflurane (1–3% isoflurane with oxygen flow rate of 1 L/min) during the surgery, and injected with carprofen (subcutaneous, 5 mg/kg), dexamethasone (intramuscular, 1 mg/kg) and lignocaine (subcutaneous at the site of incision, 3 ml/kg/h) at the start of surgery. The head was shaved around the surgery site and secured to a stereotaxic frame with ear bars and an incisor bar. An incision was made along the midline and the skull was exposed. Once bregma and lambda were identified, the skull was levelled using these landmarks. Burr holes were drilled in the skull and arrays were inserted at an angle of 8° from vertical using the following stereotaxic coordinates, such that electrodes 1 and 2 targeted the left STN: AP −4.3 to −3.8, ML +3.5, DV −9.43 mm from bregma (Paxinos and Watson, 2013; Adams et al., 2017). The implant was secured with three stainless screws placed on the skull around the head-mounted connector and acrylic dental cement, creating a head cap. Rats were monitored closely until they recovered from anaesthesia, and allowed to recover for 1 week.

### *In vivo* Electrochemical Testing

Electrochemical testing of CH-coated and uncoated Pt electrodes was performed weekly for 8 weeks. All testing was performed while the rat was awake unless otherwise stated. For all testing other than VT impedance, testing was done on electrodes 1–4 only, using electrodes 5 and 6 as reference. VT impedance was tested using the same method as *in vitro* testing described above. To determine CIL (Dalrymple et al., 2020; Shepherd et al., 2021), voltage waveform was measured (USB-6353, National Instruments, United States) in the similar manner to *in vivo* testing, but with a pulse width of 25 μs and the maximum current level of up to 2 mA to avoid any potential tissue damage and adverse behavioural response. Due to the persisting behavioural responses to CIL testing in rats in CH group, CIL testing was skipped for weeks 4, 6 and 7 for rats in this group, and weeks 5 and 8 testing was done while rats were lightly anaesthetised (1.5% isoflurane and 1 L/min oxygen) to reduce stress. In addition, while CH-coated electrodes allow for much higher CIL, to avoid adverse behavioural response due to high current level stimulation, CH-coated electrodes were not challenged beyond the CIL limit of uncoated Pt electrodes.

The parameters used *in vivo* testing were selected to suit chronic awake testing performed through the 2-cell setup. EIS and CV were measured using a potentiostat (Interface 1000E, Gamry Instruments, United States). Parameters used for EIS were frequency range of 100 Hz to 100 kHz, density of 10 points/decade and AC amplitude of 50 mV. Lower frequencies (<100 Hz) were omitted *in vivo* to avoid potential tissue damage (Dalrymple et al., 2020). For CV measurements, the same voltage range and scan rate was used as *in vitro* measurements, but with 6 cycles instead of 5 to account for increased noise resulting from movement of the animals during testing. CSC was calculated as the average of the total area of CV cycles 2–6.

### Evoked Response Recording

To test the functionality of the electrodes for neural stimulation and recording, we recorded evoked neural responses each week, after electrochemical testing. Electrodes 1–4 were used for both stimulation and recording, and electrodes 5 and 6 were used for return and reference. Stimulation was applied as a burst of 10 pulses at 80 Hz, and amplitude of up to 776 μA. Threshold for evoked responses were determined visually from averaged responses to 10 stimuli, by selecting the lowest current level that produced an evident response. Signal to noise ratio was calculated from the amplitude of evoked response at 650 μA stimulation compared to a sub-threshold stimulus.

### Histology

At the end of the 8-week timeline, rats were euthanised and transcardially perfused with 0.9% saline and 10% neutral buffered formalin (Sigma-Aldrich). Brains were dissected and post-fixed in 10% neutral buffered formalin for 2–3 days, cryoprotected in 30% sucrose in PBS and snap frozen using liquid nitrogen. Sections of 30 μm were cut and stored at −20°C freezer until sections from all rats were ready for staining. Glial fibrillary acidic protein (GFAP) and neuronal nuclei (NeuN) were chosen as markers to label glial cells and neurons. Tissue sections were first rinsed in PBS for 10 min, and incubated in blocking solution [10% normal goat serum (PCN5000, ThermoFisher) and 0.1% triton x-100 (Sigma)] for half an hour. After blocking, tissue sections were incubated in rabbit anti-GFAP (AB5804, Merck) and mouse anti-NeuN (MAB377, Merck Millipore) antibodies diluted 1:1000 and 1:200 in the blocking solution overnight at 4°C. Sections were rinsed five times in the block solution, 5 min each, and incubated in goat secondary antibodies (A11029 and A11037) diluted in PBS containing 10% normal goat serum and 0.1% tween 20 (Sigma) for 2 h at room temperature with agitation. Slides were rinsed in PBS 3 times, 5 min each. Fluorescent mounting medium (S3023, DAKO) was used to coverslip the slides. Slides were left to dry overnight, and images were taken using inverted fluorescence microscope (Observer Z1, Zeiss). Slides from all rats were immunostained at the same time, and images were obtained applying the same microscope setting.

### *In vivo* Evaluation

Out of the 16 rats implanted with a DBS electrode array, 6 rats were implanted with CH-coated electrodes (CH electrodes), and the remaining 10 rats were implanted with uncoated electrodes (Pt electrodes). Two rats implanted with Pt electrodes were used only for histology, and one was used only for VT impedance testing and histology, due to hardware failures. After implantation surgery, rats were left to recover for 1 week, and weekly electrochemical testing and evoked potential threshold testing was performed for 8 weeks.

### Image Analysis

To quantify GFAP labelling, fluorescence intensity was measured using profile plots tool in ImageJ. As fluorescence intensity was similar for all non-implanted controls (right or contralateral STN region), fluorescence measurements for implanted side in each group were compared directly.

To quantify neurons near the interface, the number of NeuN-positive cells were counted in 770 × 800 μm^2^ area which included the array interface. The process of cell counting was done manually, assisted by ImageJ’s multi-point tool to avoid double counting. As the number of cells varied depending on the slight difference in electrode targeting, the non-implanted (contralateral) side was used as a control for each brain. All statistical analyses were done using two-way ANOVA in SigmaPlot 13.

### Statistical Analysis

All *in vivo* statistical analyses were performed using SigmaPlot 13. Three-way analysis of variance (ANOVA) was used for all *in vivo* electrochemical data. Two-way ANOVA was performed to assess the effect of electrode insertion on the coating stability. The comparison between means was performed *via* Tukey’s honest significant difference criterion (Tukey-Kramer). Normality of residues was confirmed *via* Chi-square goodness-of-fit test at a confidence level of 0.05. Difference between means was regarded statistically significant with a confidence level α < 0.05. The results are stated in the format of mean ± standard deviation (SD).

## Results

### Swelling Studies and Dimensional Stability of Bulk Hydrogels

**FIGURE 3:**
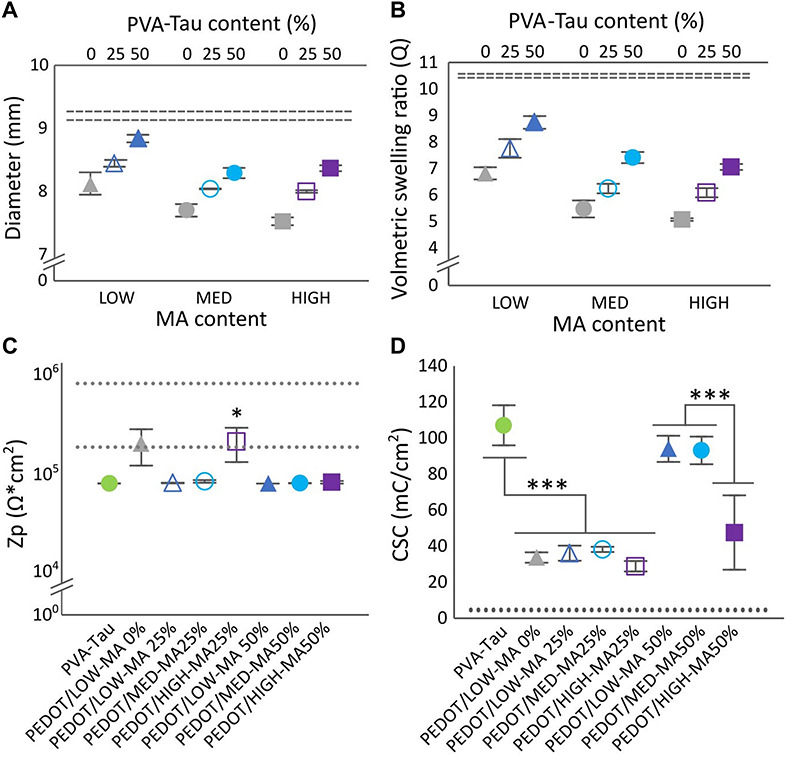
Effect of number MA groups per PVA chain and PVA-Tau content on hydrogels diameter **(A)**, and volumetric selling ratio **(B)**. Dashed lines represent the HS variant, 100% PVA-Tau 95% confidence intervals (*n* = 3, *N* = 9). **(C)** Polarisation impedance (Zp) and **(D)** charge storage capacity (CSC) of CH coated MEs. The dotted lines show the 95% confidence intervals for the Zp and CSC levels of uncoated Pt MEs. *0.05 > *p* > 0.01, ***p < 0.001. Error bars are ± 1 SEM.

The present study confirmed that dimensional stability, measured by the swelling and change in dimensions of the hydrogel, was systematically controlled by varying the crosslinking density *via* methacrylation, and doping level *via* taurine content. Methacrylate functionalisation was confirmed through proton nuclear magnetic resonance (1^*H*^-NMR, 400 MHz, D_2_O) as previously described (Nilasaroya et al., 2008). NMR analysis showed that functionalisation achieved was close to theoretical at 6, 11, and 16 for LOW, MED, and HIGH, respectively. As expected, increasing crosslinking yielded smaller dimensional changes and lower volumetric swelling as shown in Figures 3A,B.

The effect of MA and PVA-Tau content on dimensional stability was measured through changes in ø, and in the volumetric swelling ratio (Q) as shown in Figures 3A,B. Overall, three main effects were observed. First, PVA-MA hydrogels without taurine and all co-polymer formulations with 25 and 50% blended PVA-Tau had lower swelling and smaller changes in dimensions than the unblended PVA-Tau control (dashed lines in Figures 3A,B, *p* < 0.00001). The second observation was that increasing MA groups from LOW (6) to MED (11) significantly decreased ø and Q in all co-polymer formulations (*p* < 0.00001) with no further decrease in hydrogels with the highest MA density (*p* = 0.14). Finally, increasing the taurine content significantly increased ø and Q (all *p* < 0.00001) in direct proportion to the co-polymer percentage. Overall, these results show that variations in MA crosslinker groups and taurine content resulted in hydrogel systems with different swelling behaviour. All hydrogel formulations were further tested for swelling behaviour and dimensional stability as electrode coatings on MEs.

### Electrochemical Behaviour of Conductive Hydrogel Formulations as Coatings on Model Electrodes

Figure 3C shows the impedance across all the co-polymer CH formulations determined in CH-coated MEs. With exception to PEDOT/HIGH-MA 25%, all CH-coated MEs had a significantly lower impedance than Pt (Figure 3C, dashed lines, 0.01 < *p* < 0.05), comparable to that of the PEDOT/PVA-Tau 100% (positive control, *p* = 1). The impedance of PEDOT/HIGH-MA 25% overlapped that of the uncoated MEs (Pt) and the PEDOT/LOW-MA 0% (undoped control). More notable changes were identified among CH formulations upon assessing the CSC. Similar to impedance measurements, the CSC of all co-polymer variants was compared to Pt (dashed lines) and to the undoped control (Figure 3D). The assessment of CSC showed that; (1) as expected, the MEs coated with the positive control yielded significantly higher CSC levels (*p* < 0.001) than the undoped control (PEDOT/LOW-MA 0%), (2) MEs coated with 25% PVA-Tau CH formulations had similar CSC levels than the undoped control (∼34 mC/cm^2^, *p* = 0.99), irregardless of the MA content, and (3) the LOW-MA and MED-MA blended with 50% PVA-Tau (*p* = 0.87) resulted in CH coatings with CSC similar than the positive control averaging over 83 mC/cm^2^. Interestingly, the PEDOT/HIGH-MA 50% CH coating did not follow the trend of the 50% variants. The CSC level of PEDOT/HIGH-MA 50% coated MEs was significantly lower than 100% PEDOT/PVA-Tau (47.5 ± 20.6 mC/cm^2^, *p* < 0.001), with high variability overlapping CSC levels of the undoped PEDOT/LOW-MA control (*p* = 0.37).

Overall, among the co-polymers evaluated, MED-MA 50% was identified as the formulation that resulted in CH coatings with electrochemical properties comparable to the PVA-Tau reference while maintaining negligible changes in swelling degree. This new CH-coating further referred to as “LS” was engineered by blending hydrogel with an increased PVA crosslinking density, which was shown to decrease the swelling behaviour of the hydrogel coating. However, this strategy unavoidably results in a decrease in taurine moieties, which are required for doping and facilitating deposition of the conductive polymer PEDOT. Thus, here we show that despite the decrease in doping density, the LS CH has equivalent electrical properties than the previously reported highly conductive, HS variant.

### Effect of Electrode Insertion on the Conductive Hydrogel Coating Stability

**FIGURE 4:**
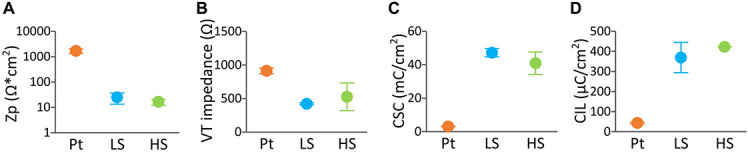
Baseline electrochemical properties of DBS arrays uncoated (*n* = 4), LS CH-coated (*n* = 2) and HS CH-coated (*n* = 2) under equilibrium swelling conditions. The measurements represent the average of the arrays (each array is the average of four electrode sites). Electrochemical testing regime consisted of **(A)** polarisation impedance (Zp), **(B)** voltage transient (VT) impedance, **(C)** charges storage capacity (CSC), and **(D)** charge injection limit (CIL) determined with a 100 μs biphasic, charge-balanced pulse **(D)**. Error bars are ± 1 SD.

Initial *in vitro* tests assessed the electrochemical performance of DBS arrays coated with LS or HS coatings compared to uncoated Pt arrays. As shown in Figures 4A–D, the baseline Zp, VT impedance, CSC, and CIL in, DBS arrays displayed enhanced electrochemical properties compared to Pt for both CH coatings (Zp, *p* < 0.01, VT impedance, 0.01 < *p* < 0.05, CSC, *p* < 0.0001, CIL, *p* < 0.001). Specifically, the CH coatings had over 2 orders of magnitude lower Zp and approximately twofold decrease in VT impedance. This was coupled with 1 to 2 orders of magnitude higher CSC, and over ninefold increase in CIL. No significant differences in electrochemical properties were observed between the two different CH formulations (Zp, *p* = 0.99, VT impedance, *p* = 0.589, CSC, *p* = 0.22, CIL, *p* = 0.34). In addition, no differences were observed in the electrical properties of Pt arrays before and after insertion in the agarose gel (results not shown), indicating that the agarose gel is not likely to influence the electrochemical measurements.

**FIGURE 5:**
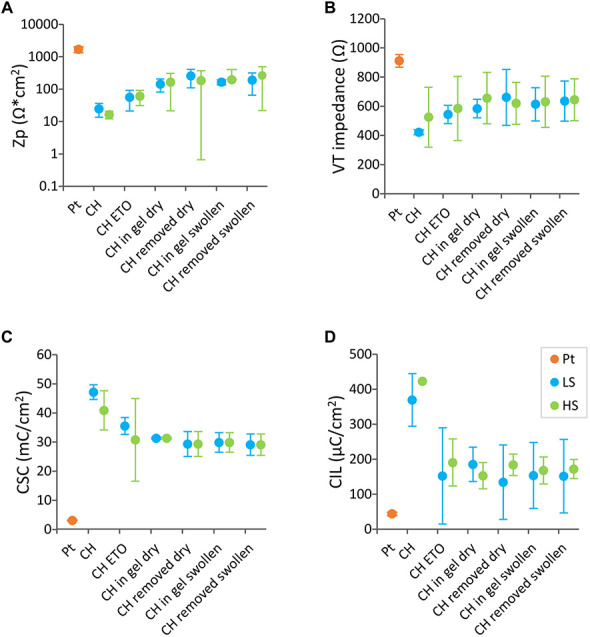
Electrochemical properties of CH-coated electrode arrays in different conditions. Pt electrode arrays (*n* = 4) were CH-coated with the low swelling (LS) formulation (*n* = 2) (blue) or with the high swelling (HS) reference formulation (*n* = 2) (green). Each data point represent the average of arrays (each array is the average of four electrode sites). **(A)** polarisation impedance (Zp), **(B)** voltage transient (VT) impedance, **(C)** charges storage capacity (CSC), and **(D)** charge injection limit (CIL). The electrochemical properties were assessed following ethylene oxide sterilisation, (ETO) then upon insertion in agarose gels with the CH coating dry (Inserted Dry). Arrays were removed, assessed (Removed) and then inserted with the CH coating swollen (Inserted Swollen) in a new agarose gel and removed for a final test.

Figure 5 shows the electrochemical properties of DBS arrays CH-coated with the LS or the HS formulation throughout the insertion process showing the effect of swelling condition of the coating during insertion (swollen or dry). Four main effects were identified; (1) electrical properties were not affected upon insertion (CSC, *p* = 0.79, VT impedance, *p* = 0.59, Zp = 0.19, CIL, *p* = 0.99), which was the case for both coating swelling conditions, (2) no significant changes were observed in any impedance metric due to CH coating type (Zp, *p* = 0.89 or VT impedance, *p* = 0.59) or due to insertion condition (Zp, *p* = 0.19 or VT impedance, *p* = 0.6), (3) With regards to CSC, both CH coatings maintained similar CSC values, however at the last testing conditions the CSC values were significantly lower than those prior to ETO sterilisation (0.01 < *p* < 0.05). (4) Lastly, a significant decrease was observed in CIL of both CH coatings after ETO sterilisation (0.01 < *p* < 0.05).

The insertion and removal of DBS arrays was recorded to macroscopically assess the effect of electrode insertion on CH coating stability (videos included in Supplementary Material). LS arrays (dry or swollen) and HS arrays (dry) were not obviously affected during insertion or removal. One key limitation though, inherent to all coatings during the fabrication process, was that the dip-coating technique often resulted in non-uniform hydrogel coating across the array (see Supplementary Videos). This was shown by the formation of blebs across the array, interspaced by thinner regions, an effect referred to as “beading” that was more pronounced in the HS variant. In addition, the DBS array with a HS swollen coating presented an excess hydrogel build-up in between the return electrodes and the working electrodes that upon insertion resulted in detachment and displacement of the coating over the return electrodes (see Supplementary Video 1). While this did not affect the electrical properties of the unaffected working electrodes, it did raise concerns on the suitability and appropriateness to implant the HS coated electrodes in the rat DBS model. Therefore, the LS formulation with dry insertion was chosen for use in *in vivo* study, and from here on, LS is referred to as CH.

### Conductive Hydrogel Coating Improves Electrochemical Performance of Rat Deep Brain Stimulation Electrodes

**FIGURE 6:**
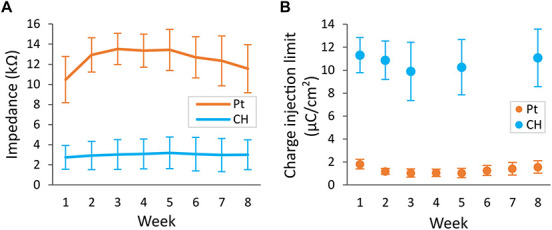
Voltage transient (VT) impedance and charge injection limit (CIL) measured in the rat brain. Conductive hydrogel-coated electrodes (CH) or uncoated platinum electrodes (Pt) were implanted in rat brains and electrochemical testing was performed weekly for 8 weeks. The maximum current that can be applied before reaching the potential for water reduction (E_mc_ = –0.6 V) was measured as CIL. VT impedance was calculated from the peak voltage in the first phase of response to fixed current of 100 μA delivered at 100 μs pulse width. Both graphs represent mean ± SD. **(A)** VT impedance was significantly lower in CH compared with Pt (*p* < 0.001, three-way ANOVA, 36 CH electrodes, 48 Pt electrodes) across all electrode contacts for the duration of the study. There was significant effect of duration of implantation in Pt (*p* < 0.001), increasing in the first 2 weeks and decreasing in the final 2 weeks. **(B)** CIL was significantly higher in CH compared with Pt (*p* < 0.001, three-way ANOVA, 24 CH electrodes, 28 Pt electrodes) across all electrode contacts for the duration of the study.

Conductive hydrogel-coated electrodes had significantly lower VT impedance (3.0 ± 1.5 kΩ, 36 electrodes) compared with Pt electrodes (12.5 ± 2.3 kΩ, 48 electrodes), across all electrode contacts at all time-points (Figure 6A, *p* < 0.001, three-way ANOVA). VT impedance of Pt electrodes was significantly affected by the duration of implantation (*p* < 0.001), increasing in the first 2 weeks and decreasing in the final 2 weeks, whereas that of CH electrodes remained stable throughout. Due to the detectable behavioural response to CIL testing in the CH group, rats in the CH group were tested only at weeks 1, 2, 3, 5, and 8 (weeks 5 and 8 under anaesthesia). The CH group had significantly higher average CIL (10.57 ± 2.21 μC/cm^2^, 24 electrodes) compared with Pt electrodes (1.30 ± 0.49 μC/cm^2^, 28 electrodes) in all electrode contacts throughout the study (Figure 6B, *p* < 0.001, three-way ANOVA), with no evidence of a reduction over the study duration.

**FIGURE 7:**
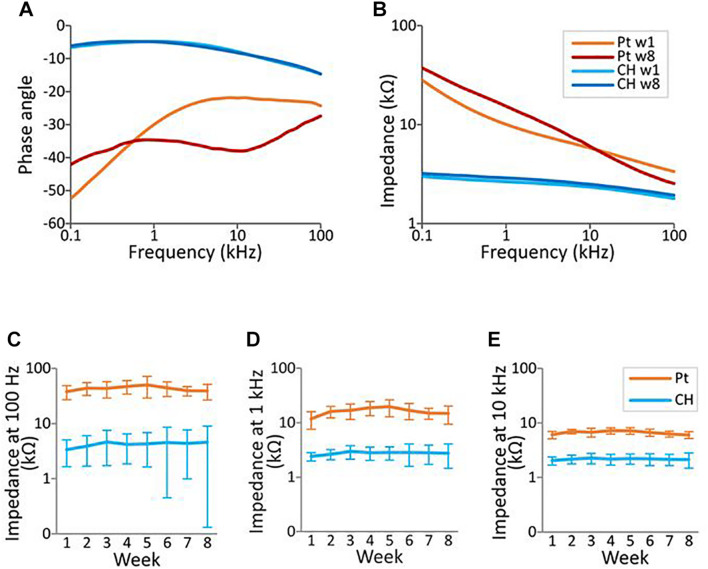
Electrochemical impedance spectroscopy (EIS) measurement in the rat brain. EIS was measured within the frequency range from 100 Hz to 100 kHz using a potentiostat. **(A,B)** Representative impedance and phase angle measurement of conductive hydrogel-coated (CH) and uncoated platinum (Pt) electrodes at the start (w1, week 1) and end (w8) of chronic implantation. **(C–E)** All graphs represent mean ± SD. CH electrodes had significantly lower impedance at 100 Hz, 1 kHz, and 10 kHz throughout the duration of the study (*p*’s < 0.001, three-way ANOVAs, 28 Pt electrodes, 24 CH electrodes). There was no significant effect of duration of implantation or electrode contacts at all three frequencies (*p*’s > 0.4).

Representative electrochemical impedance spectroscopy traces for both electrode groups are shown in Figures 7A,B. CH-coated electrodes had significantly lower impedance, measured *via* electrochemical impedance spectroscopy, compared with Pt electrodes at 0.1, 1, and 10 kHz (all *p* < 0.001, three-way ANOVA) across all electrode contacts, throughout the whole duration of implantation (Figures 7C–E). Duration of implantation had no significant effect on the impedance at any of the three frequency levels tested (*p* > 0.5).

**FIGURE 8:**
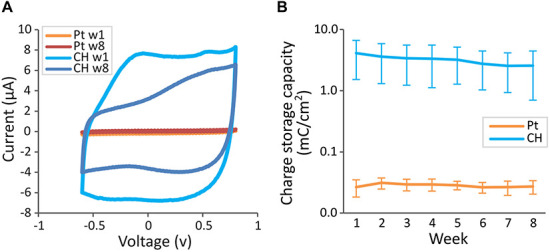
Charge storage capacity (CSC) of conductive hydrogel-coated (CH) and uncoated platinum (Pt) electrodes tested weekly in the rat brain. Cyclic voltammetry was measured between –0.6 and 0.8 V for six cycles using a potentiostat. CSC was calculated as the average area inside the cyclic voltammetry traces. **(A)** Representative cyclic voltammetry traces of CH and Pt electrodes at the start (w1) and the end (w8) of chronic implantation. **(B)** Mean charge storage capacity ± SD measured *in vivo*. CH had significantly higher charge storage capacity (*p* < 0.001, three-way ANOVA, 28 Pt electrodes, 24 CH electrodes) across all the electrode contacts for the duration of the study. There was no significant effect of duration of implantation (*p* = 0.106), or interaction between coating and duration of implantation (*p* = 0.108).

Representative cyclic voltammetry traces for both electrode groups are shown in Figure 8A. As apparent in the greater areas within the CV traces for CH electrodes, CH electrodes had significantly higher CSC (3.20 ± 2.10 mC/cm^2^, 24 electrodes) compared with Pt electrodes (0.028 ± 0.007 mC/cm^2^, 28 electrodes) (Figure 8B, *p* < 0.001). Both CH and Pt electrodes had stable CSCs, which were not affected significantly by the duration of implantation (*p* = 0.106).

### Conductive Hydrogel Coating Does Not Affect Evoked Potential Threshold Recordings

**FIGURE 9:**
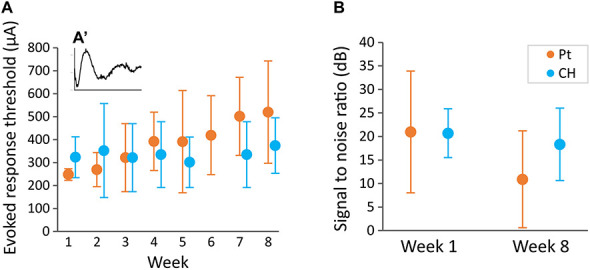
Evoked potential threshold measured weekly and signal to noise ratio at the start and end of the study. Rats implanted with DBS electrode arrays with conductive hydrogel-coated (CH) or uncoated platinum (Pt) electrodes were stimulated with burst of 10 pulses at 80 Hz at different current levels (100–650 μA) to record evoked responses **(A′)**. Graphs represent mean ± SD. **(A)** Evoked response threshold was not significantly affected by CH coating throughout the duration of the study (*p* = 0.206, two-way ANOVA, 3–4 rats for Pt and CH). There was no significant effect of duration of implantation (*p* = 0.470), or interaction between coating and week (*p* = 0.636). **(B)** Signal to noise ratio was calculated from the amplitude of evoked response at 650 μA stimulation compare to a sub-threshold stimulus. Signal to noise ratio was not significantly affected by CH coating (*p* = 0.443, two-way ANOVA, 7 rats for Pt, 4 rats for CH). There was no significant difference between start and end signal to noise ratio (*p* = 0.189).

To test the effect of CH-coating on the functionality of the electrodes for neural stimulation and recording, EP recordings were performed weekly. A typical EP is illustrated in Figure 9A′ insert. There was no significant difference in EP threshold (minimum current required to produce a clear response) between CH (333 ± 122 μA) and Pt (378 ± 161 μA) groups (*p* = 0.272). Despite an apparent trend for Pt thresholds to increase over time, there was no significant effect of the duration of implantation (*p* = 0.443, Figure 9A). Additionally, there was no significant difference in signal to noise ratio between CH and Pt at the start (20.69 ± 5.20 dB vs 20.98 ± 12.94 dB, 4 CH rats, 7 Pt rats) or at the end (18.33 ± 7.7 vs 10.90 ± 10.31, 4 CH rats, 7 Pt rats) (*p* = 0.443, Figure 9B).

### Histological Analysis of Tissue Response and Neural Survival

**FIGURE 10:**
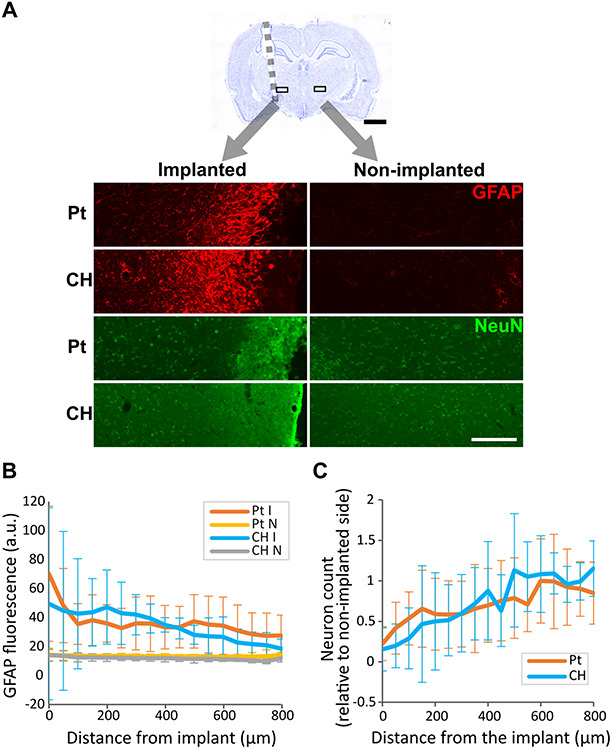
Tissue response and neural survival at the site of implantation in the rat brain implanted with conductive hydrogel-coated (CH) or uncoated platinum (Pt) electrodes. The brains were sectioned and immunostained for glial fibrillary acidic protein (GFAP) and neuronal nuclei (NeuN) to assess tissue response and neural survival (10 rats for Pt and 6 rats for CH). All graphs represent mean ± SD. **(A)** Representative images of GFAP and NeuN immunostaining in the rat brains implanted with Pt and CH electrodes. Scale bar, 200 μm. **(B)** GFAP fluorescence was significantly higher near the implant (*p* < 0.001), but was not significantly affected by CH coating (I, implanted; *N*, non-implanted; *p* = 0.387, two-way ANOVA). **(C)** The relative number of NeuN positive cells (neurons) to non-implanted were significantly reduced near the implant compared to the area further away from the implant (*p* < 0.001), but was not significantly affected by CH coating (*p* = 0.352, two-way ANOVA).

To determine the effect of CH-coating on tissue response and neural survival, at the completion of the 8-week study, rats were sacrificed, and brain histology was analysed. Tissue response and neural survival was visualised with immunofluorescence staining using GFAP and NeuN antibodies (Figure 10A). As expected, tissue response to all implants was significantly higher near the electrode (Figure 10B, *p* < 0.001, two-way ANOVA, 10 Pt rats, 6 CH rats); however, there was no significant difference between Pt and CH groups (*p* = 0.387). Similarly, the number of neurons (NeuN-positive cells) were significantly reduced near the implant compared to the area further away from implants (Figure 10C, *p* < 0.001, two-way ANOVA, 10 Pt rats, 6 CH rats), but there was no difference between the groups (*p* = 0.352).

**FIGURE 11:**
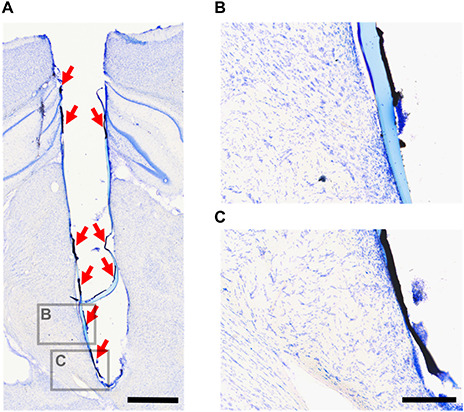
Delaminated CH coating in the rat brain. After tissue fixation and electrode array removal, the brains were sectioned and stained with cresyl violet. This is an example of delaminated CH coating remaining in the brain after electrode array removal (**A**, red arrows). Higher magnification images **(B,C)** show that the CH coating delaminated in large pieces. Scale bar **(A)** = 1 mm (applies to **A**), scale bar **(B)** = 200 μm (applies to **B,C**).

Delamination of CH coating was observed in 2 out of 6 rats implanted with CH-coated electrodes after implant removal. One of these rats had a loose head cap, and complete delamination of CH coating that was left in the brain after removal of the array (Figure 11A). The head cap on the other rat was stable, however, the implant was removed and re-inserted during surgery due to targeting difficulties. In this rat, delaminated CH coating was found near the tip of the electrode track after removal of the array. In both of these rats, the CH coating delaminated in large pieces (approximately 0.1–0.6 mm long), and degradation into smaller pieces was not observed (Figures 11B,C).

## Discussion

This study showed that CH coatings tailored with low swelling properties improve the electrochemical performance of smooth Pt electrodes in the brain without causing significant alterations in neural stimulation/recording functions of the electrodes, or in the tissue response or neural survival at the electrode-tissue interface. We found CH-coated electrodes showed significantly lower VT impedance and EIS, and higher CIL and CSC compared with uncoated smooth Pt electrodes in the rat brain, and these changes were maintained for the entire 8-week duration of the study. Thresholds for evoked response and signal-to-noise ratios were not significantly altered due to CH-coating. There was also no significant difference in GFAP immunoreactivity or number of NeuN-positive cells at the electrode-tissue interface between CH-coated and uncoated Pt electrodes.

### Tailoring Swelling Behaviour of Conductive Hydrogel Electrode Coatings

In chemically crosslinked hydrogels, increasing the number of functional groups has been reported to increase crosslinking density (Canal and Peppas, 1989; LaPorte, 2017), which translates into lower degrees of swelling. Here we show that the volumetric swelling (Q) decreased when hydrogel formulations increased in MA content from 6 (LOW) to 11 (MED) but not from 11 (MED) to 16 (HIGH). As previously reported, the chemical modification with high degrees of functional groups can lead to an increased chance of macromer cyclisation that reduces crosslinking efficiency (Anseth et al., 1996; Martens et al., 2007; Nafea et al., 2011), which was expected to influence the ability of HIGH-MA hydrogels to create tighter networks. However, a more significant effect was observed as a function of the taurine content. Taurine contains a sulfonate group that conveys the molecule with high electronegativity, a key property required for doping like PEDOT and allows its growth through the hydrogel network. In this study, a consistent trend was observed where the ø and Q, increased in direct proportion to the PVA-Tau content in the hydrogel formulation, even with a high degree of MA functionalisation. These results are consistent with literature reports showing that the incorporation of ionic groups in a polymeric matrix can influence swelling behaviour (Pinchuk et al., 1984; Okay et al., 1998; Wu et al., 2004; Zhou et al., 2014). The incorporation of highly charged moieties in the PVA backbone has been suggested to create substantial electrostatic force repulsion between the anionic groups, which results in increased swelling, interfering with the crosslinking reaction, and limiting the polymerisation efficiency. As described by Goding et al. (2017), attaching 40 taurine functional groups to a PVA backbone can result in mass swelling ratios as high as 250.

The taurine content and the network volume available for growing PEDOT were identified as potential restricting factors influencing the overall electrochemical performance. First, the 25% PVA-Tau co-polymers produced CH coated MEs with decreased electrical performance compared to the 50% CH counterparts with LOW and MED crosslinking degree and with less confidence to the higher crosslinked variants. It is known that in PEDOT-coated electrodes the CSC increases in direct proportion to the amount of PEDOT deposited (Cogan, 2008; Antensteiner and Abidian, 2017). In CH systems, PEDOT deposits through the hydrogel network *via* the taurine groups immobilised to PVA chains (Goding et al., 2017). It is possible that the taurine density in 25% co-polymers may not allow for a continuous, widespread growth of PEDOT thus resulting in lower CSC levels.

In addition to the taurine content, a size-restricted hydrogel network (as determined through Q evaluation) could affect the ability of growing PEDOT, resulting in suboptimal electrical performance. This was observed in PEDOT/HIGH-MA 50% CH coatings, which resulted in CSC levels overlapping those observed in the 25% CH variants, despite having similar taurine content than the LOW and MED 50% variants. Similar to the effect of MA content on swelling behaviour, this could be associated to macromer cyclisation (a phenomenon likely to occur in formulations with high degrees of functional groups), which could be impacting on the network permeability to allow even and efficient PEDOT growth.

### Effect of Electrode Insertion on the Conductive Hydrogel Coating Stability *in vitro*

Delamination of electrode coatings has been previously reported as a limitation of material technologies that aim to improve the electrical and biological performance at the neural interface (Green et al., 2012a; De Faveri et al., 2014; Dalrymple et al., 2020). In this study, the assessment of baseline properties confirmed that both low and high swelling CH coatings variants have equivalent electrical properties, which are superior to uncoated Pt arrays. In addition, insertion or removal from agarose gels did not significantly affect the electrical stability of the CH-coated arrays, regardless of the degree of swelling of the CH coating formulation. However, ETO sterilisation was found to significantly impact the electrical properties, resulting in increased impedance and decreased charge injection levels. It is possible that ETO reacts with the amine groups present within the tethers of the functional groups of PVA within the CH coatings (Goding et al., 2017), an effect that has been observed in collagen gels resulting in decreased stability of the collagen structure (Van Luyn et al., 1995). Further research is required to fully elucidate the effect of ETO sterilisation on CH coatings.

The hydrogel beading effect was an additional issue that caused uneven distribution of the coating across the array, although it did not affect the stability of the coatings as measured in the *in vitro* model. This beading was observed in most arrays regardless of the CH formulation but was more pronounced in the HS variant. In fact, the hydrogel beading between working and return electrodes caused CH coating detachment on the return electrodes (most proximal) in one of the HS coated arrays when inserted swollen. This issue and the associated degree of swelling was not observed in the LS coated arrays. Given that the beading issue may contribute to partial loss of coating into the tissue previously reported in the higher swelling variant (Dalrymple et al., 2020), the lower swelling CH-coating was selected for *in vivo* studies. Overall, the *in vitro* agarose model may not be ideal for evaluating CH coating stability.

### Stability of Conductive Hydrogel Coating *in vivo*

While the electrochemical performance of CH-coated electrodes was stable during the duration of the study, delamination of low swelling CH was detected in 2 out of the 6 rats in this study. In our recent study using a higher swelling CH formulation, we reported CH delamination in 4 out of 5 rats implanted with CH electrodes in the cochlea (Dalrymple et al., 2020). In that study it was hypothesised that the delamination was caused by the process of removing the array post-termination, as the electrochemical performance of the CH electrode arrays remained stable throughout the study. In the current study, the electrochemical performance of the CH-coated electrodes also remained stable throughout the duration of the study. As the removal of electrode arrays involves drilling and removing the skull around the dental acrylic head cap, some movement of the array during this process is inevitable. It is however difficult to determine at what point CH coating became delaminated in this study, as the pressure in the brain tissue may hold the delaminated CH close to the implant, maintaining good electrochemical performance while the implant is in situ. Analogue studies have improved the mechanical stability of hydrogel electrode coatings by roughening (plasma etching) the metallic substrate of recording microwires (De Faveri et al., 2014). Although surface roughening can be readily translated to Pt-based DBS arrays, this technique is limited to the metallic sites of the array and has not been tested at a scale comparable to a DBS insertion. Furthermore, maintaining the integrity of the coating across the array is of relevance for using the CH as vehicle for drug delivery.

One of the rats had complete delamination of CH from its implant. A distinguishing feature of this rat was that it had a loose head cap where dental acrylic cement lifted from the skull slightly. Due to this loose head cap, micro-movement of the array inside the brain during connecting and disconnecting cables was likely to have been greater than normal. The electrochemical performance of the implant was affected only in electrode 1 (the closest to the tip), especially in the final 3 weeks of the study. In the other rat that exhibited delamination, some pieces of CH were found in the brain tissue near the tip of the electrode track after implant removal. This rat did not have a loose head cap but is the only rat that had the implant re-implanted during surgery for correcting neuronal targeting. Notably, the electrochemical performance of this implant was not affected.

In comparison, no CH residues were observed in the agarose studies (results not shown). However, unlike the implantation process, electrode arrays tested *in vitro* were removed carefully but without the assistance of a stereotaxic frame. In addition, tissue fixation that occurred prior to the electrode array removal could have affected mechanical properties of the brain tissue. Ultimately, both factors were likely to add unintended mechanical stress during electrode removal leading to coating delamination, consistent with previous observations (Dalrymple et al., 2020). These examples suggest that delamination of CH used in this study is likely to be rare for standard successful surgery and removal of CH-coated arrays implanted in the brain. Removal and re-insertion of CH-coated electrodes may reduce the stability of CH coating, and therefore would not be appropriate in a clinical setting. Further improvement in stability of CH by using different formulations or manufacturing approaches may be beneficial in such situations.

### Effect on Neural Stimulation and Recording

In studies using microelectrodes to record multiunit activity and single unit response (Ludwig et al., 2006; Baranauskas et al., 2011; Harris et al., 2013; Viswam et al., 2019), lower impedance electrode coatings resulted in improved signal-to-noise ratio. In the present study, using larger electrodes to record evoked potentials, no significant difference in signal-to-noise ratio between CH and Pt groups was observed, despite the significant difference in impedance. This lack of improvement in signal-to-noise ratio is likely due to the large surface area electrodes already providing a sufficiently low impedance that the signal-to-noise ratio is dominated by the biological background, such as spiking activities of distant neurons, rather than electrode noise. Similarly to signal-to-noise ratio, threshold for eliciting evoked response was not significantly affected by the CH coating. This was in agreement with the previous report by Dalrymple et al. (2020), where evoked auditory brain response threshold did not differ significantly between CH-coated and uncoated electrodes despite significant electrochemical improvement in CH-coated electrodes.

It should be noted that the rat DBS electrodes used in this study (diameter of about 0.4 mm near STN), while smaller than DBS electrodes (diameter of 1.3 mm), are still proportionally larger when compared to the STN volume. Preliminary testing of the CIL of these electrodes with the highly conductive CH coating produced severe adverse effects on the rats when delivered during the chronic awake testing, thus it was avoided. The current study was not designed to demonstrate an improvement in stimulation or recording of the CH coated electrodes *in vivo*, but rather to demonstrate the long-term *in vivo* stability of the new CH coating in an application requiring the coating to be in direct contact with neural tissue. To demonstrate the functional advantage of CH coating, it will be necessary to utilise the CH coated electrodes in situations requiring higher charge concentration such as in smaller, directional DBS electrodes (Dembek et al., 2017; Anderson et al., 2019), and for recording smaller neural signals such as local field potential as feedback signal for closed-loop stimulation (Cagnan et al., 2019; Tinkhauser et al., 2019).

### Tissue Response

After the initial acute inflammatory phase, the mechanical mismatch between metal electrodes and soft target tissue such as the brain has been one of the issues raised as one of the factors affecting chronic tissue response for traditional metal electrodes (Gulino et al., 2019). Soft and flexible implants are expected to cause less tissue damage compared to more rigid implants (Weltman et al., 2016; Lee et al., 2017). The CH coating is expected to reduce mechanical mismatch of metal electrodes to neural tissues, and therefore reduce tissue response as the result (Green and Abidian, 2015; Spencer et al., 2017). Our recent *in vivo* study however, suggests that the foreign body response to CH is greater than that to the traditional metal electrodes (Dalrymple et al., 2020). This was attributed to possible loss of coating into the tissue. In the current study, examining lower swelling variants of the CH coating, coated electrodes did not cause an increased tissue response compared to uncoated Pt electrodes after 8 weeks of chronic implantation. The CH coating may have been expected to produce less trauma; however, trauma associated with insertion and array dimensions have been shown to dominate the tissue response (Villalobos et al., 2020). Thus, it is possible that the proportional electrode size is outweighing any improved tissue response associated with enhanced coating mechanics.

As we observed increased tissue response in CH (HS) group compared to Pt group in the previous study (Dalrymple et al., 2020), the similar level of tissue response observed in CH (LS) group compared to Pt group in the present study may indicate the improvement of stability in LS compared to HS. Furthermore, the chemical versatility of the PVA hydrogel component is a key advantage of this CH coating technology since it can be loaded with drugs such as neural growth factors or anti-inflammatory agents (Aregueta-Robles et al., 2014; Green and Abidian, 2015). Loading CH with correct drugs may help reduce tissue response and improve neural survival, outperforming uncoated Pt electrodes.

### Future Study

This study demonstrated the electrochemical advantage of CH coating on Pt electrodes in the brain. This was a passive *in vivo* study, with no chronic stimulation. In addition, although much higher CIL was available for CH-coated electrodes, due to the behavioural response to high current level stimulation, electrodes were not challenged beyond the CIL limit of uncoated Pt electrodes. Incorporating smaller electrode designs to utilise the higher charge injection capacity will be required for future studies. With the knowledge that reduction in mechanical mismatch alone did not reduce tissue response in the brain, it would also be beneficial to test whether loading the CH with drugs can improve tissue response and neural survival in a future study.

The significant electrochemical advantage and stability of CH in the brain indicates the potential of using CH coating for designing smaller, higher fidelity electrode arrays for more specific neural stimulation for improvement of chronic neurological conditions.

## Data Availability Statement

The raw data supporting the conclusions of this article will be made available by the authors, without undue reservation.

## Ethics Statement

The animal study was reviewed and approved by the St Vincent’s Hospital Animal Research Committee (project ethics #010/19 and #009/20) and complied with the Australian Code for the Care and Use of Animals for Scientific Purposes (National Health and Medical Research Council of Australia) and the Prevention of Cruelty to Animals (1986) Act.

## Author Contributions

LP-W and JF developed the protocols, conducted the analysis, contributed to writing the manuscript, and coordinated the study. TH developed the protocols, conducted the animal experiments and analysis, and led writing the manuscript. UA-R developed the protocols, conducted *in vitro* experiments and analysis, and contributed to writing the manuscript. WA and JV contributed to animal experiments and analysis and writing the manuscript. WD performed acquisition, interpretation and data analysis of hydrogel swelling studies and electrical performance of coated model electrodes and contributed to writing the manuscript. All authors contributed to the article and approved the submitted version.

## Conflict of Interest

The authors declare that the research was conducted in the absence of any commercial or financial relationships that could be construed as a potential conflict of interest.

## Publisher’s Note

All claims expressed in this article are solely those of the authors and do not necessarily represent those of their affiliated organizations, or those of the publisher, the editors and the reviewers. Any product that may be evaluated in this article, or claim that may be made by its manufacturer, is not guaranteed or endorsed by the publisher.
